# Investigation of COVID-19 Impact on the Food and Beverages Industry: China and India Perspective

**DOI:** 10.3390/foods10051069

**Published:** 2021-05-12

**Authors:** Shafique Ul Rehman Memon, Vijayanta Ramesh Pawase, Tushar Ramesh Pavase, Maqsood Ahmed Soomro

**Affiliations:** 1 College of Management, Ocean University of China, Qingdao 266100, China; 2 Institute for Technology and Management (ITM) Business School, Navi Mumbai 410210, India; 3 College of Food Science and Engineering, Ocean University of China, Qingdao 266003, China; tusharpavase@yahoo.com; 4 College of Marine Life Science, Ocean University of China, Qingdao 266003, China; soomroamaqsood@gmail.com

**Keywords:** COVID-19, global pandemic, food and beverages industry, sustainable supply chain, China, India

## Abstract

The sudden breakout of coronavirus disease (COVID-19) rapidly spread across the globe, leaving no country behind in being affected by the global pandemic in the year 2019–20. As COVID-19 commenced, within months two major Asian giants initiated the norms of social distancing and lockdowns in their societies. The indiscriminate nature of the current pandemic has not only impacted the health and quality of life of people but has also disrupted the global economy, supply chains, and countries all over the world. In food and beverage manufacturing industries, the unanticipated disruption has encumbered its lock on the global food supply chain and service sector as major cities shut down for several months in China and India. Human existence is dependent upon food, which renders energy for activity, growth, and all functions of the body. Although both China and India have shown eminent response to tackle the ongoing pandemic, the food supply chain remains vastly exposed to significant COVID-19 risks. This research primarily investigates the ongoing COVID-19 scenario in two major economies (China and India), delivering insight into the pandemic’s impact within the food and beverage manufacturing sectors, and explores the policies adopted and strategies undertaken for sustainability in food supply chains.

## 1. Introduction

**Figure 1:**
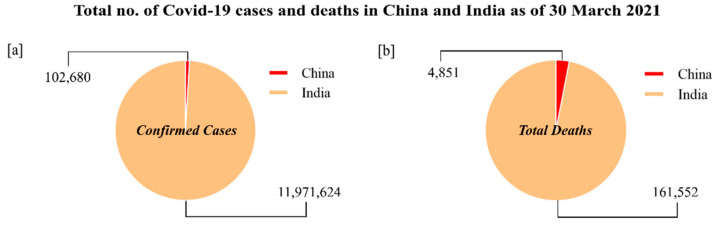
(**a**) COVID-19 confirmed cases and (**b**) deaths in China and India, 30 January 2021 (data adopted from WHO COVID-19 dashboard).

In the past two decades, the world has encountered various virus-related epidemics, for instance severe acute respiratory syndrome coronavirus (SARS-CoV) 2002–03, H1N1 influenza (2009), Middle East respiratory syndrome coronavirus (MERS-CoV) 2012, Zika 2016 and severe acute respiratory syndrome coronavirus-2 (SARS-CoV-2) 2019–20 [1,2]. In late December 2019, a new pneumonia outbreak originated in the largest metropolitan area of Wuhan in the Hubei province of China [3]. The Chinese Center for Disease Control and Prevention (CDC) concluded that the first unexplained case of new pneumonia emerged on 12 December 2019 as non-SARS nCoV. However, on 11 February 2020, the World Health Organization (WHO) announced a new name for the epidemic disease caused by 2019-nCoV, coronavirus disease (COVID-19), and the International Committee on Taxonomy of Viruses (ICTV) renamed the formerly termed 2019-nCoV as severe acute respiratory syndrome coronavirus-2 (SARS-CoV-2) [4]. At present (30 March 2021), there are 126,359,540 COVID-19 confirmed cases reported in the world with total 2,769,473 deaths and 102,765,148 recovered. In China, there are 102,680 confirmed cases with total 4851 deaths and they are presently facing a second wave of COVID-19; however it remains under control with proper implementation of testing and individualized health QR codes used to gauge the path of the virus and contain the outbreak [5]. Contrarily, India has been severely affected by COVID-19 with 11,971,624 confirmed cases and 161,552 deaths (WHO Coronavirus Disease (COVID-19) dashboard) (Figure 1a,b). Currently, India is going through a crisis mainly due to the ongoing second wave of COVID-19, which has resulted in a significant increase in the infected number of cases and deaths compared to the previous wave. Lockdowns and restrictions have been imposed once again. Nevertheless, there is a large proportion of recovered patients from COVID-19 at the same time.

Major economies are already shifting the dynamics within global supply chains [6] and significant parts of supply chains are now located in various major economies. According to 2019 data, the 16 major economies contribute approximately 78% of the global Gross Domestic Product GDP of USD 86.31 trillion, which is attributable to the trillion-dollar club. The top five major economies in terms of nominal GDP are the U.S., China, Japan, Germany and India, which add 55% to the total GDP of the world [7].

The COVID-19 outbreak has affected supply chains and has attracted the attention of researchers [8] and business experts [9] all over the world. Moreover, the impact of the pandemic on society and economy has been witnessed due to the lockdown of cities all over the world, labor mobility restrictions, travel bans, airline suspensions, and most importantly slowdown of the economy, which is seriously influencing the sustainability of supply chains in many businesses around the globe [10].

Among many manufacturing sectors, the food and beverage industry is one of the excelling industries, through which basic needs for human development are fulfilled [11,12]. Since food is a fundamental requirement for human nourishment, normal operations should be sustained to feed the people [13] during pandemic-like situations. On average, terrestrial agriculture provides 83% of the food consumed by humans [14]. The food industry is a dynamic, global network of diverse businesses that provide the majority of the world’s food. In 2020, a report revealed that the food and beverage industry is one of the fastest growing in the world [15].

In view of COVID-19′s new social, environmental and economic landscapes, a variety of businesses are dealing with various problems of a barricaded world with a certain degree of losses [16]: in particular, production losses, deterioration of supply chains, cancelation of export orders, shortage of raw materials and obstruction of transport, among others [17,18]. Newly released data have found that 94% of Fortune 1000 firms are experiencing volatility related to COVID-19, whereas 75% have been adversely affected [19]. The global GDP is projected to decline from 2.3% to 4.8% [20]. According to the report of the International Civil Aviation Organization in 2020 [21], the activities of the supply chain remain significantly affected by the COVID-19 pandemic mainly in the food processing industry, tourism and hospitality, education, fashion and apparel, leather and other retail sectors.

The food and beverage industry in two major Asian giants i.e., China and India, has faced several challenges due to COVID-19, which include supply chain operations, safety, training, disaster management and responses, awareness, re-forming business models, digitalization and advancement of technology, and other unanticipated impacts. In addition, the consumer behavior towards food has been extensively changed in both nations [22,23]. Therefore, the ongoing impacts of COVID-19 and the future of the food and beverage industry in China and India need precise examination for better understanding of the recovery and responsive nature of operations toward COVID-19-like unforeseen events.

From an operations perspective, there is a high risk of emergency in food and beverage industry due to unanticipated events. In spite of various food safety and monitoring boards in both nations, there is a high necessity in the food and beverage industry to institute health and safety protocols on an individual level. In addition, there is a high scope of reformation in the supply chain operations to produce risk-free food and beverage products in a safe, financial, and environmentally sustainable manner.

Thus, this research conducts an in-depth assessment of the impact of the COVID-19 pandemic on China and India’s food and beverage industries and provides qualitative and quantitative research through graphical representations. The study mainly focuses on: the food and beverage industry in China and India; COVID-19 impact; digitalization and technological advances in food and beverage industry during the pandemic; strategies, policies and responses of China and India during COVID-19; and finally outlining the future of China and India’s food and beverage industry post-pandemic, respectively.

### Problem Statement and Research Objectives

A pandemic such as COVID-19 has not only affected the smooth operations of food supply chains but also resulted in food insecurity conditions in several nations. Since the beginning of COVID-19, a variety of research has been carried out on the impact of the pandemic on different supply chains; however, no study has been carried out concerning the impact of COVID-19 on the food and beverage industry in Asian countries. Thus, the country where the first COVID-19 patient was identified, i.e., China, and the second-largest COVID-19-affected country, i.e., India, were selected in the present research. The current study aims to investigate the impact of COVID-19 on the food and beverage industry in China and India through the research objectives (RO) given below:

RO1: What is the scenario of the ongoing pandemic in China and India?
RO2: What is the scale of research in different fields concerning COVID-19 carried out during the pandemic and ongoing in both nations?
RO3: What is the scale of research on food and beverage commodities concerning COVID-19 in both nations?
RO4: What is the impact of COVID-19 on the food and beverage industry in both nations concerning the following factors: loss of enterprises, growth in revenue (%), total profit (%), gross value added and growth value of exports (%)?
RO5: Did COVID-19 bring a sudden change in consumer food behavior, digitalization, and technology advancement in both nations?
RO6: Which strategies, policies, and responses were undertaken against COVID-19 for smooth operations of food supply chains by China and India?
RO7: What is the future of China and India’s food and beverage industries post-pandemic?

## 2. Materials and Methods

In the present study, both quantitative and qualitative methodologies were utilized to investigate the COVID-19 impacts on the food and beverage industry in China and India. The data were collected for a period of five years (2016 to 2020) and formulated into graphical representation using GraphPad Prism Version 8.4.3 (graphing and statistics software), respectively. To create graphs, Prism is widely used in social sciences and many other fields [24,25,26,27]. Our graphical analysis revealed rich and clear results [28], which were further used for examining the impacts of COVID-19 on Food and Beverage (F&B) industry (prior to COVID-19, during COVID-19 and ongoing in the COVID-19 period) concerning various factors mentioned in RO4. The negative and positive impacts of COVID-19 on the F&B industry in China and India were then addressed in Section 4.1 and Section 4.3, respectively. Furthermore, literature and data searches were obtained using the keywords: COVID-19; global pandemic; COVID-19 in China and India; food and beverages manufacturing industry in China and India; COVID-19 impact on food and beverages industry in China and India; and COVID-19 and sustainability.

All the information obtained was mainly from articles published on PubMed, Sciencedirect, and Google scholar. Reports were taken from various webpages such as the World Health Organization (WHO) COVID-19 dashboard, World Bank, Food and Agricultural Organization (FAO) and International Monetary Fund (IMF). Country-specific websites, National Bureau of Statistics of China, China Commercial Industry Research Institute, Food Safety and Standard Authority of India and Ministry of Statistics and Program Implementation (MOSPI), Federation of Indian Chambers of Commerce and Industry (FICCI), etc. English and Chinese articles were included; data extraction was carried out according to the above-specified terms and COVID-19-related information. The obtained key findings from this research were summarized, investigated, and then the consensus was made.

## 3. Results

### 3.1. Research on COVID-19 in China

**Figure 2:**
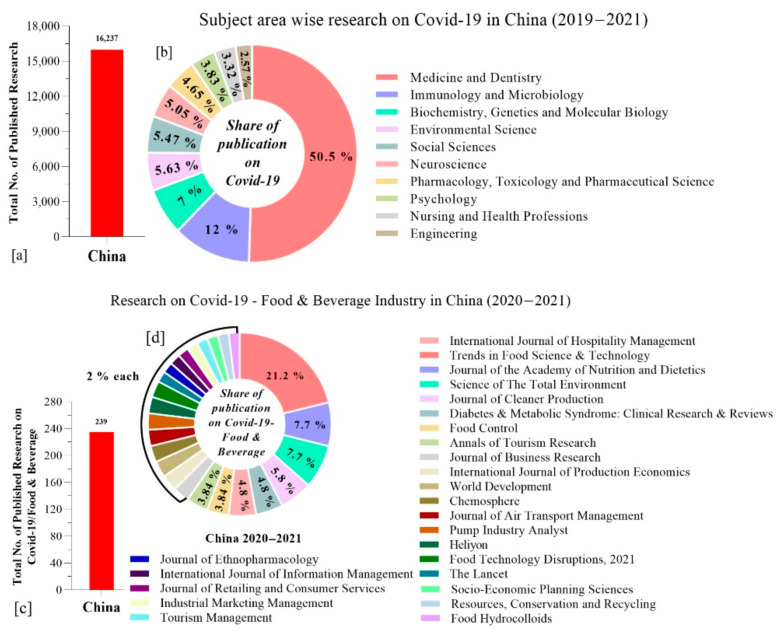
(**a**) Total number of published research studies on COVID-19; (**b**) subject area specific research on COVID-19 in China (2019–2021); (**c**) total no. of research studies and (**d**) research publications on COVID-19/food and beverage sector in China (2020–2021).

As the first case of COVID-19 was detected in mid-December 2019, numerous researchers initiated their studies on COVID-19 in different fields focusing on China. During 2019–2021 (10 February), the total number of research studies carried out on COVID-19/the global pandemic in various fields was approximately 16,237 (Figure 2a) in which the share of publications in the field of medicine and dentistry was observed to the highest (50.5%), followed by immunology and microbiology (12%), and biochemistry, genetics and molecular biology (7%) (Figure 2b).

The research studies carried out specifically concerning COVID-19/food and beverages in China totaled 239 during 2020–2021 (10 February) (Figure 2c,d). All this research was not limited to only COVID-19-related food security and safety, but also involved environmental, social, and economic studies.

### 3.2. Research on COVID-19 in India

**Figure 3:**
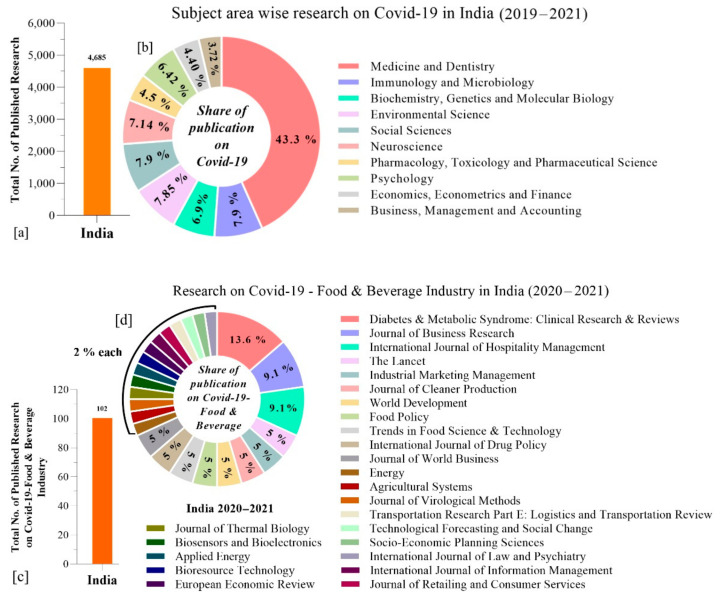
(**a**) Total number of published research studies on COVID-19; (**b**) subject area specific research on COVID-19 in India (2019–2021); (**c**) total no. of research studies and (**d**) research publications on COVID-19/food and beverage sector in India (2020–2021).

The first case of COVID-19 in India was found on 27 January 2020. Compared to China, numerous studies were also carried out on COVID-19/global pandemic in India, however, only 4,685 research publications were determined in 2019–2021 (10 February) (Figure 3a). Amongst various fields, medicine and dentistry constituted 43.3%, followed by: immunology and microbiology (7.9%); social sciences (7.9%); environmental science (7.85%); neuroscience (7.14%); biochemistry, genetics and molecular biology (6.9%) and psychology (6.42%) (Figure 3b).

The research carried out specifically concerning COVID-19/food and beverages in India totaled 85 during 2020–2021 (Figure 3c,d). All this research was not limited to only COVID-19-related food security and safety, but also involved environmental, social, and economic studies.

### 3.3. Food and Beverage Industry in China

China is home to roughly one-fifth of the world’s population and one of the world’s fastest-growing consumer markets, providing a significant boost to the food and beverage industry [29,30,31]. China’s population and economy have been changed as a result of dramatic expansions in industrial capability and growing consumer consumption [32,33]. Over the last 40 years, China’s food and beverage consumption has changed significantly, shifting from an industry-driven to a consumption-driven economy. The speed at which customer tastes and bargaining power are changing has been the driving force behind this transition. In addition, as consumers’ disposable income and desire to spend more grows, new categories and brands emerge [34].

Since 2018, China’s food consumption has steadily progressed into the era of experience economy, in which consumers put a higher importance on the need to consume food and beverages for delicacy and pleasure and are willing to pay more for goods that meet this need. The aim of drinking beverages has changed over time from basic needs such as thirst quenching to physiological and psychological needs such as work, taste, and differentiation. Changes in demand are causing the supply to shift. Soda retailers have accelerated research and development, with major brands introducing new products with features including bubbles, probiotics, and slimming qualities that quickly met rich taste and blending requirements in 2018. Meanwhile, mixed-taste beverages have been rapidly gaining market share. The beverage industry has transformed because of evolving consumer tastes and manufacturers’ willingness to adapt rapidly with new technologies to meet these needs. This has enabled manufacturers to encourage their customers to seek multi-functional goods for which they are willing to pay a higher price [34].

**Figure 4:**
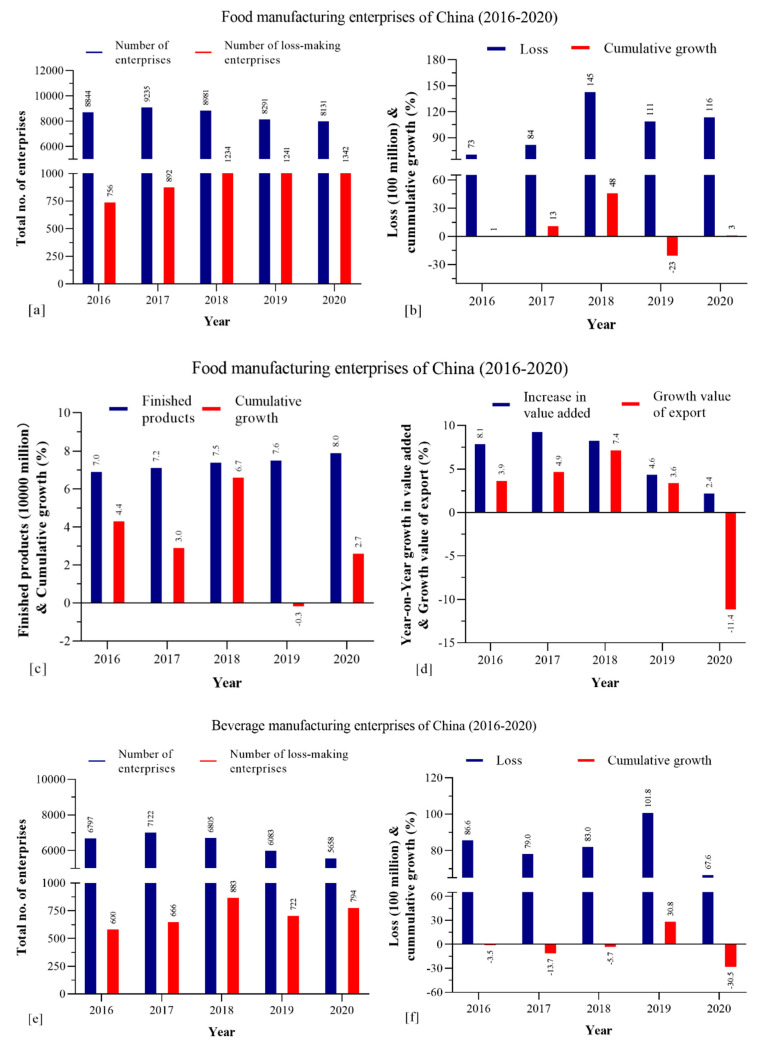

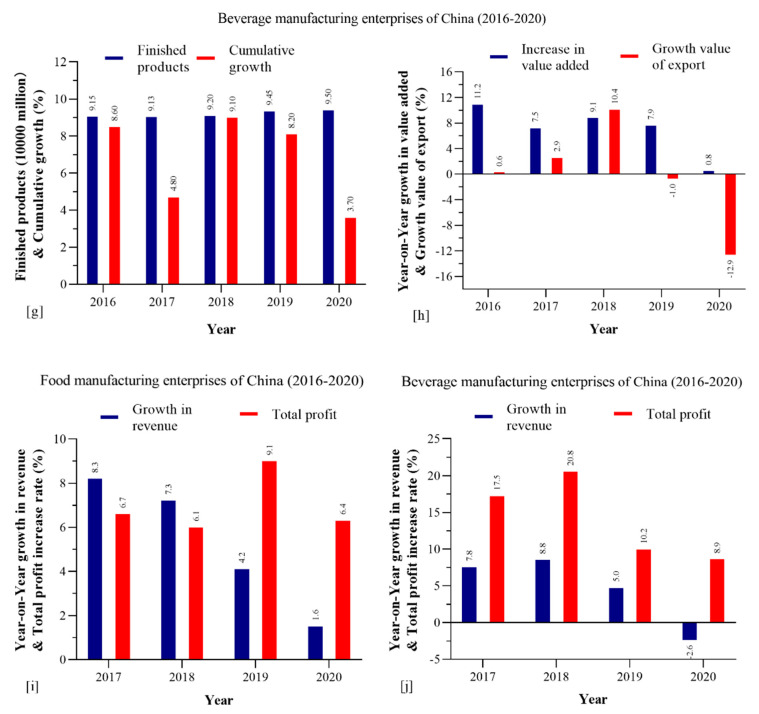
(**a**) Food manufacturing enterprises and loss-making enterprises in China. (**b**) Loss and cumulative growth (%). (**c**) Finished products and cumulative growth (%). (**d**) Year-on-year growth in value added and growth in value of export (%). (**e**) Beverage manufacturing enterprises and loss-making enterprises in China. (**f**) Loss and cumulative growth (%). (**g**) Finished products and cumulative growth (%). (**h**) Year-on-year growth in value added and growth in value of export (%). (**i**) Food and beverage manufacturing year-on-year growth in revenue and (**j**) total profit increase rate (%).

The Annual Report on Catering Industry Development of China 2019 released in June found that China is the second-largest food and beverage economy in the world in revenue with the U.S. at number one, and is also the world’s largest importer of food and beverages [35]. In 2017, China imported 53.48 million tons of food and beverages with total value worth USD 58.28 billion, reflecting a 36.5% and 25% year-on-year increase, respectively. The average annual growth rate of China’s imported food trade has remained at 5.7% over the last five years, from 2013 to 2017. According to the International Trade Administration (ITA), the food and beverage sector reached approximately USD 595 billion in 2019, a 7.8% increase over 2018 [36] which is projected to reach USD 176,857 m in 2021 with 16% growth [37]. According to the National Bureau of Statistics, there are a total of 8131 food and 5658 beverage registered processing enterprises in China [38]. In 2018, the registered units of food and beverages totaled 15,786, however the number declined to 13,789 in 2020 (Figure 4a,e).

This must be due to the impact of COVID-19, where the loss-making enterprises in the food industry were 1234 in 2018, which increased to 1241 in 2019 and 1342 in 2020 (Figure 4a). On the contrary, in the beverage industry there were 883 loss-making enterprises in 2018, however this declined to 722 in 2019 and 794 in 2020 (Figure 4e). The reason behind this could be the change in consumer behavior witnessed during the COVID-19 pandemic. In recent years, with the continuous and steady growth of the national economy, the continuous improvement of residents’ consumption levels and the upgrading of consumption structure, China’s beverage industry has shown new fluctuations. In terms of output, China’s total beverage output has fluctuated since 2014. From 2016 to 2018, the national beverage output declined and it rebounded in 2019; the national beverage output in 2019 was 177,634,800 tons, a year-on-year increase of 7%. As of January–May 2020, China’s beverage production reached 59.99 million tons, a year-on-year decrease of 12.6% [39]. Hence, there was a slight increase in the beverage demand, which resulted in decreasing the loss-making enterprises during 2019 and 2020, respectively.

China’s food manufacturing enterprises’ cumulative growth dropped to −23% in 2019, then rebounded to 3% in 2020 (Figure 4b). In 2019, the food manufacturing loss was reduced from China Yuan (CNY)145 billion to CNY 111 billion, but due to the COVID-19 impact, the loss increased to CNY 116 billion in 2020 (Figure 4b). On the contrary, the beverage industry’s cumulative growth was consistently in a loss, estimated at −3.5% in 2016, −13.7% in 2017, and −5.7% in 2018. However, there was a remarkable gain in the cumulative growth in 2019 at 30.8% which severely dropped by −30.5% in the year 2020; this could be due to the COVID-19 impact (Figure 4f).

The cumulative growth for finished goods in the food manufacturing industry dropped to −0.3% in 2019 and increased to 2.7% in the 2020 (Figure 4c). In addition, the year-on-year growth in gross value added (GVA) and growth value of export both dropped to 4.6% and 3.6% in the year 2019, which further severely declined to 2.4% and −11.4% in 2020, respectively (Figure 4d). In the beverage industry, the cumulative growth for finished goods dropped from 9.10% to 8.20%, and drastically dropped further to 3.70% in 2020 (Figure 4g). In addition, the year-on-year growth increase in the gross value added (GVA) and growth value of export both dropped to 7.9% and −1% in 2019, which severely dropped again to 0.8% and approximately −13%, respectively (Figure 4h).

The year-on-year growth in revenue and total profit increase rate percentage were investigated for the food and beverage industry in China. The year-on-year growth in revenue reduced from 7.3% in 2018 to 4.2% in 2019; however, the total profit increased from 6.1% in 2018 to 9.1% in 2019. In 2020, both growth in revenue and total profit increase rate dropped to 1.6% and 6.4%, which certainly could be due to the impact of COVID-19 (Figure 4i).

In the beverage manufacturing industry, the year-on-year growth in revenue was reduced from 8.8% in 2018 to 5% in 2019; and the total profit increase rate reduced from 20.8% in 2018 to 10.2% in 2019. Owing to the COVID-19 impact, both year-on-year growth in revenue and total profit increase rate further dropped to −2.6% and 8.9% in 2020 (Figure 4j), respectively.

**Figure 5:**
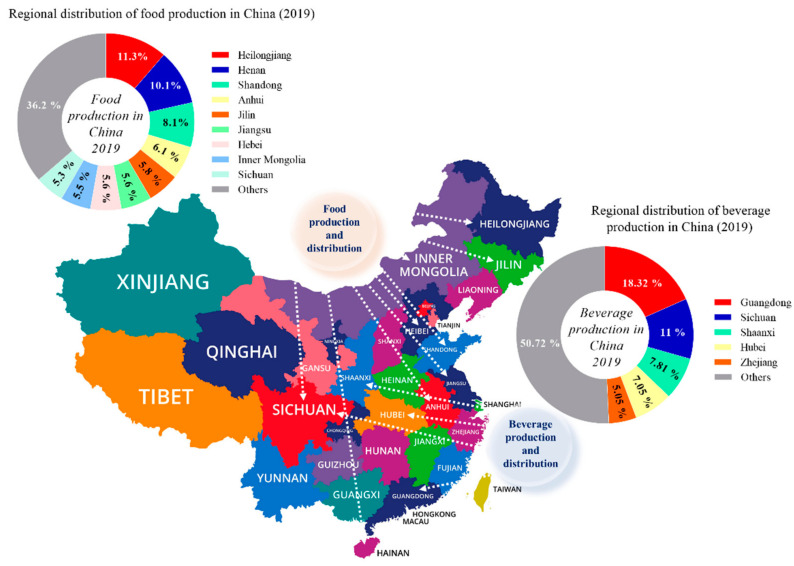
Regional distribution of food and beverage manufacturing industries in China.

The major food producing industries and their distributions are located in various provinces of China (2019), for instance the Heilongjiang province is the topmost food manufacturing province in China contributing a share of 11.3%, followed by Henan 10.1%, Shandong 8.1%, Anhui 6.1%, Jilin 5.8%, Jiangsu and Hebei 5.6%, Inner Mongolia 5.5%, Sichuan 5.3%, and others 36.2%, respectively (Figure 5).

The major beverage production industries and their distributions are from the Guangdong province, ranking topmost by contributing 18.32% of the total share of beverage production in China, followed by Sichuan 11%, Shaanxi 7.81%, Hubei 7.05%, Zhejiang 5.05%, and others 50.72% in 2019, respectively (Figure 5).

**Figure 6:**
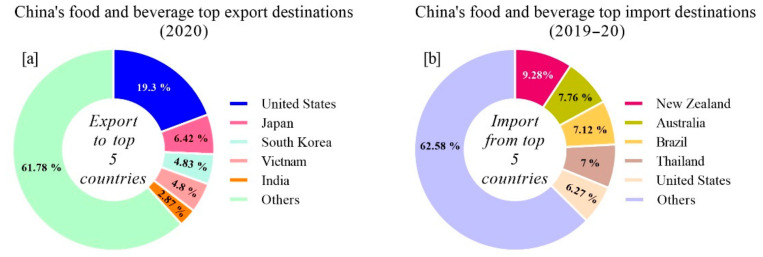
China’s topmost food and beverage (**a**) export and (**b**) import destinations in the world.

In the year 2020, the major export and import destinations for food and beverage products in China were as follows: China’s top five export destinations for food and beverage were the United States, comprising 19.3% of the total exports, followed by Japan 6.42%, South Korea 4.83%, Vietnam 4.8%, India 2.87%, and others 61.78%, respectively (Figure 6a).

On the other hand, China’s top five import destinations for food and beverages included New Zealand as the topmost nation, contributing 9.28% of the total imports, followed by Australia 7.76%, Brazil 7.12%, Thailand 7%, United States 6.27%, and others 62.58%, respectively (Figure 6b).

**Figure 7:**
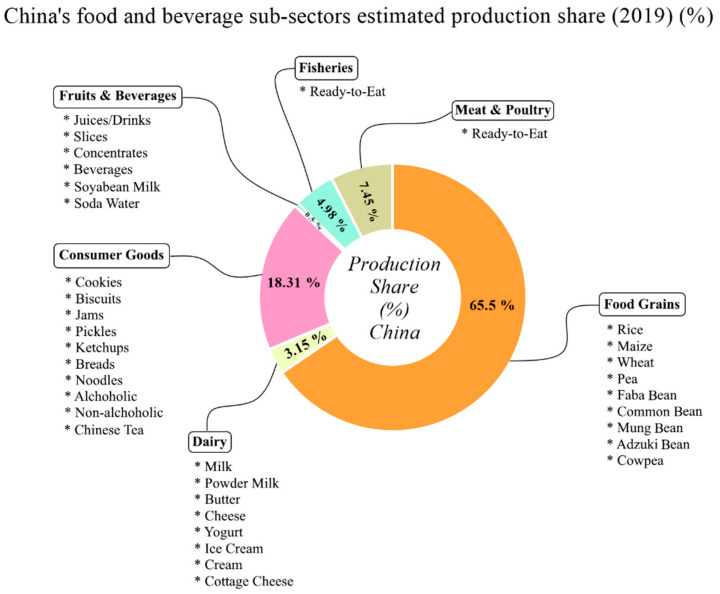
China’s food and beverage sub-sectors and estimated production share (%).

It is noteworthy that China’s food and beverage sub-sectors estimated share of production (2019) are categorized into mainly food grain 65.5% as the top sub-sector, followed by consumer goods 18.31%, meat and poultry 7.45%, fisheries 4.98%, dairy 3.15% and fruits and beverages 0.5%, respectively (Figure 7). The food and beverage products in the sub-sectors are classified according to their high production and demand, for instance the food grains include rice, maize, and wheat as the major produces. Consumer goods comprise cookies, biscuits, noodles, pickles, Chinese herbal tea, and alcoholic and non-alcoholic drinks. Dairy products include liquid milk, powdered milk, cheese, ice cream, yogurt, cottage cheese, and tofu. Seafood and meat products mainly comprise processed and ready-to-eat products, respectively (Figure 7).

### 3.4. Food and Beverage Industry in India

According to a report produced by the Confederation of Indian Industry (CII) and Grant Thornton, India is projected to become the world’s fifth-largest consumer market by 2025. The largest consumption category is food and beverages. The vast agriculture sector in the country supports the food and beverage sector. India is the world’s largest producer of pulses and the world’s second-largest producer of rice, wheat, sugarcane, and fruits and vegetables. It is also the world’s largest producer of milk and buffalo meat, as well as the fifth-largest producer of poultry. Other beneficial factors include vast areas of arable land, a pleasant climate, a long coastline, and low wages. Company barriers were lowered after the economy was liberalized in the early 1990s. With the introduction of new retail structures such as supermarkets, the sector has gained more access to the market. Meanwhile, logistics for transportation and storage have changed. Other benefits for the industry include a large population (1.366 billion in 2019) and a growing middle class [40].

Around half of the population in India is under the age of 30 and many of them begin working early in order to improve their quality of life [41]. Consumers with higher income levels have more disposable income. Families’ lifestyles have shifted, and more are dining out and trying new cuisines. Convenience foods are becoming more common among working couples. Consumers have become more discerning, and those who live in urban areas put a higher emphasis on branded foods because they guarantee consistency.

A segment of the population has become increasingly health conscious [42]. This market is shifting away from carbohydrate- and fat-rich foods and toward protein-rich foods, fruits, and vegetables. Customers who trust consistency have driven the bottled water industry to USD 50 million. The beverage industry is worth around USD 16 billion, excluding alcoholic drinks [43]. The most popular beverages are tea and coffee, followed by soft drinks (carbonated beverages and juices), health drinks, milk-based beverages, flavored beverages, and energy drinks. Unpackaged tea and coffee account for half of all tea and coffee consumed in the world. The global market for alcoholic beverages is estimated to be worth USD 35 billion, with whisky, beer, and wine being the most common beverages [40,44].

**Figure 8:**
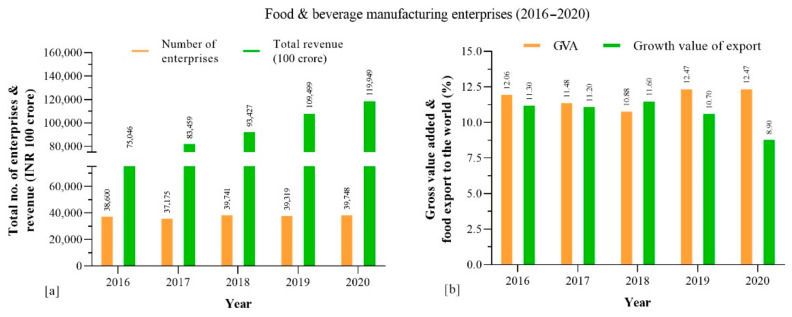
Food and beverage manufacturing enterprise in India (2016–2020). (**a**) Total number of enterprises and total revenue (INR 100 crore). (**b**) Gross value added and growth of export (%).

India’s total number of registered food and beverage manufacturing enterprises remained consistent from 2018 to 2020 with slight changes in their numbers. The total revenue generated from the food and beverage industry in India significantly increased from 75,046 (100 crore Indian Rupees (INR)) to 119,949 (100 crore INR), (1 USD = 72.45, 28 March 2021), respectively (Figure 8a).

The gross value added and growth value of export remained almost consistent in 2016 to 2017; however there was a significant increase observed of 12.47% in 2019. Furthermore, growth value of export declined from 11.60% in 2018 to 10.70% in 2019, and again dropped to 8.90% in 2020; this could be due to the impact of COVID-19 in the country (Figure 8b).

**Figure 9:**
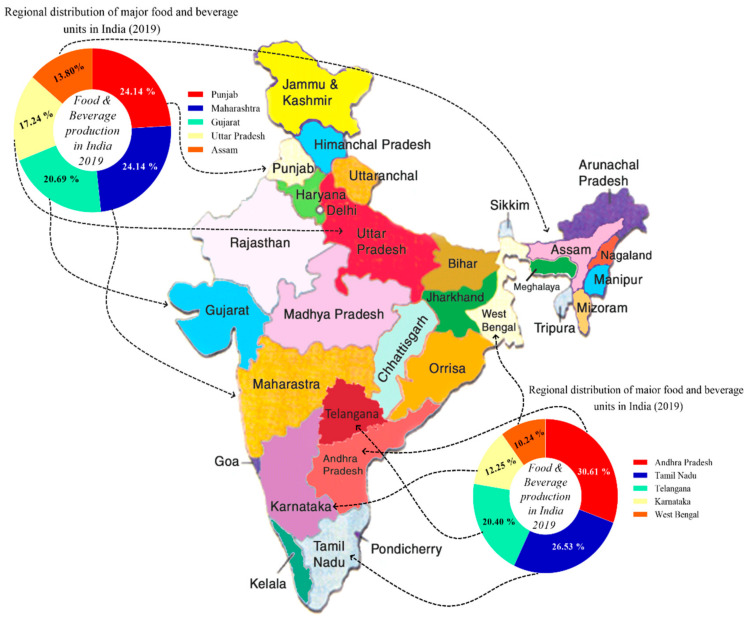
Regional distribution of food and beverage manufacturing industries in India.

The major food producing industries and their distributions are located in various provinces of India. Thus, the top food and beverage producing industries were categorized into different regions of India. In the northern and western part of India, Punjab and Maharashtra ranked first with 24.14% of production share, followed by Gujarat 20.7%, Uttar Pradesh 7.24%, and the Northeastern side of India i.e., Assam province, with 13.80%, respectively (Figure 9).

In the southern regions of India, Andhra Pradesh ranked first with more than 30% of the total production and distribution of food and beverage products, followed by Tamil Nadu 26.53%, Telangana 20.40%, Karnataka 12.25%, and West Bengal 10.24%, respectively (Figure 9).

**Figure 10:**
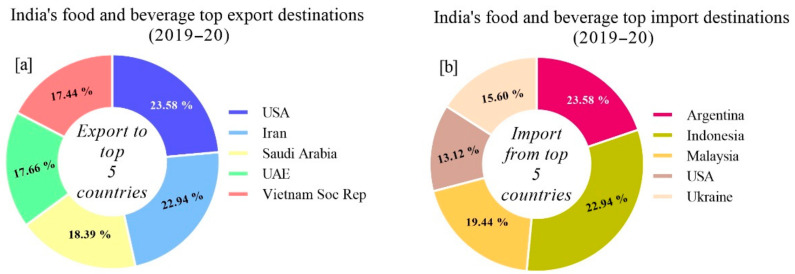
India’s topmost food and beverage (**a**) export and (**b**) import destinations in the world.

In the year 2019–2020, the major export and import destinations for food and beverage products in India were as follows: India’s top five export destinations for food and beverage were United States comprising 23.58% of the total exports, followed by Iran 22.94%, Saudi Arabia 18.39%, United Arab Emirates 17.66%, and Vietnam 17.44%, respectively (Figure 10a).

On the other hand, India’s top five import destinations for food and beverage included Argentina 23.58%, Indonesia 22.94%, Malaysia 19.44%, Ukraine 15.60%, and United States 13.12%, respectively (Figure 10b).

**Figure 11:**
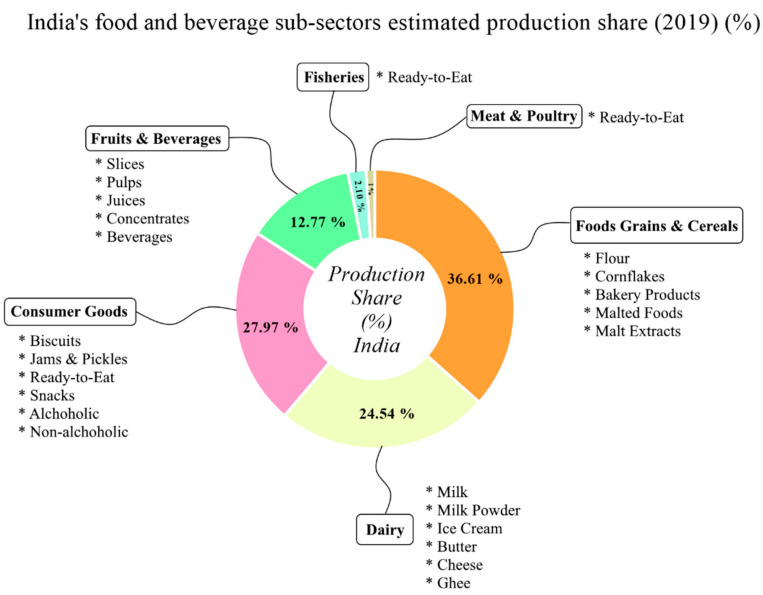
India’s food and beverage sub-sectors and estimated production share (%).

Of note, India’s food and beverage sub-sectors estimated shares of production are categorized into mainly food grain and cereals as the top sub-sector at 36.61%, followed by consumer goods 27.97%, dairy 24.54%, fruits and beverages 12.77%, fisheries 2.1%, and meat and poultry comprising 1%, respectively (Figure 11).

The food and beverage products in the sub-sectors are classified according to their high production and demand, for instance the food grains include wheat, flour, cornflakes, bakery products, malted foods, and malt extracts as the major products. Consumer goods comprise biscuits, jams, pickles, snacks, and alcoholic and non-alcoholic drinks. Dairy products include liquid milk, powdered milk, ghee, butter, cheese, ice cream, yogurt, and cottage cheese. Seafood and meat products mainly comprised processed and ready-to-eat products, respectively (Figure 11).

## 4. Discussion

### 4.1. Impacts of COVID-19 on Food and Beverage Industry in China and India

Many agricultural industries around the world have witnessed serious impacts of the COVID-19 pandemic, including two Asian major economies, China and India [45,46,47,48,49,50,51,52,53,54]. The countries affected by COVID-19 were compelled to rethink and amend their food and agriculture strategies in order to ensure that public food sources remained sufficient and accessible.

According to a United Nations survey, food malnutrition mounted from 23.3% to 26.4% from 2014 to 2018. In addition, due to the outbreak of COVID-19 in September 2019, nearly 821 million citizens, or > 10% of the world’s total population, are facing hunger [55]. It was reported that in the end of 2020, the COVID-19 pandemic could impact the food supplies of another 260 million people [56,57]. Approximately 135 million people in the 55 low- and middle-income countries experienced food insecurity before the pandemic. However, several integrated calamities, such as COVID-19, locust attacks, ongoing climate changes and armed conflicts, have resulted in vulnerability of the food supply [58].

During the pandemic, a major focus was drawn on the essential components of the food commodities concerning availability, accessibility, utilization, and stability [59]. The logistic, distribution, and delivery obstructions were the main factors for unstable food supply hindering access during the pandemic [60,61]. In addition, due to the ongoing tariff and trade affairs, import and export were severely affected which resulted in disruption in food stock availability [61,62]. Such conditions were highly evident in China and India as revealed in Figure 4d,h, where both countries reported declining trends in their imports and exports, particularly in 2020.

Of note, an approximately 17% reduction in the domestic and foreign volume of trades was witnessed due to overall factory terminations in China as per the reports accessed on “Xinhua net” between the period of January and February 2020 [63]. Nations across the planet must reassure a long-term food reserve to meet global demands. COVID-19 economic shocks not only shortened the availability of labors but also disrupted the normal cultivation of crops and industrial production, which resulted in postponements in the continuous supply [59]. Figure 4e,g reveals the decline in the cumulative growth in finished goods in China. Recently, several research studies have empirically demonstrated that economic shocks are propagating. A research by You et al. [64] showed that the direct economic losses in the transportation, logistics, warehousing, postal, food, and beverage service industries of China amount to CNY 21.61 billion, and the monthly indirect economic losses are CNY 36.4 billion for all industries. During lockdown, the gross monthly economic losses hit CNY 177.04 billion. Moreover, the domestic supply chains of China have suffered detrimental effects. Transactions between domestic firms have decreased by 60%, whereas orders between international firms have decreased by 50% [63]. The domestic food supply has also been damaged by huge wastage of agricultural products that were unable to be shipped out due to the government’s implementation of movement restriction controls [65,66].

Just as COVID-19 affected China’s food industry, India was not spared from the economic shocks as well, and currently some regions of India are still severely affected by the pandemic. Major disruptions in domestic food supply were caused by labor shortages, logistic bottlenecks, and income losses, which resulted in immediate impacts on the domestic food supply and availability [67,68]. A tragic incidence in the Indian state of Karnataka’s Chikkaballapur district reported a farmer dumping a total of six harvesters of grapes on the roadside due to disruption caused by COVID-19 [69]. Many farmers have drained their crops into pits due to a lack of transportation services. The grape farmers alone are likely to lose around Rs 500–600 crores (USD 68–82 million) in the Chikkaballapur and Bengaluru rural districts. Similarly, since there was no way to sell milk during the lockdown, a milk seller in Karnataka’s Belagavi district dumped 1500 L of milk into a canal [69,70]. Furthermore, it was revealed in China and India that retail shortages are exacerbated by a temporary scarcity at store supply chains caused by desperation buying by consumers [71], which appears to have been quickly resolved at almost all supermarkets with stabilized supply.

The food supply chain generally includes production, post-harvest storage, packaging, distribution/retail, and, ultimately, dinner plates, all of which pose virus transmission risks. As a result, during the current crisis response, maintaining a secure food supply, customer and worker health, and effective food delivery has become a significant task [72]. Farmers and small- to medium-sized businesses in developing countries are being hit hard by the pandemic, which is exacerbated by a lack of advanced IT and transportation infrastructure. [73]. Transportation and shipping activities were difficult as a result of movement restrictions and lockdowns; food companies that import raw ingredients suffered the most. Since local producers and small food supply chains are less limited by lockdown legislation, improving and providing technological solutions to them proved to be an effective strategy for alleviating the crisis.

Both Chinese and Indian governments are working towards sustaining the current situation by implementing new regulations to meet the food production and associated supply chain needs in line with the increasing demands [74,75].

### 4.2. Food and Beverage Monitoring Safety Authority Organizations of China and India

**Figure 12:**
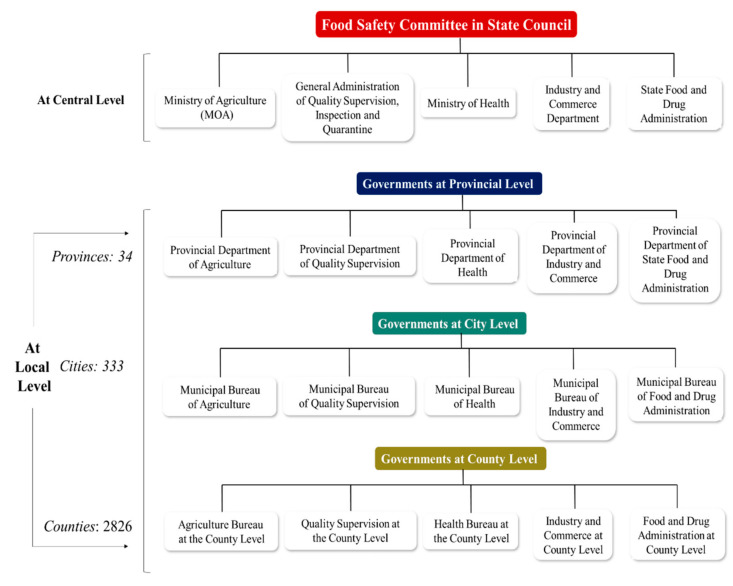
Food safety monitoring organizational structure of China.

In China, the central governments Ministry of Health (MOH), Ministry of Agriculture (MOA), General Administration of Quality Supervision, Inspection and Quarantine (AQSIQ), Industry and Commerce Department (IAC) and State Food and Drug Administration (SFDA) are in charge of administrative management, but not of inspection services that have direct interaction with the food industry, trade, or the public, as specified in the People’s Republic of China’s Constitution (1982). Local governments are subordinate to the central government and are responsible for implementing laws, according to the Constitution. Article 5 of the FSL, which assigns responsibility for food safety oversight and administration at the county level or higher, sets out the accountabilities for food safety at the local government level. This provides that the executive departments of each government section’s regulatory responsibility (e.g., health, agriculture, quality management, industry and trade, and food and drug administration) must follow this Law and the State Council Regulations. At the local level, there is a hierarchy of three levels of government that are active in the food safety management system, which are provinces, districts, and counties, and each level reports to the level above it. Figure 12 depicts the relationship between departmental oversight and administration at all levels [76].

**Figure 13:**
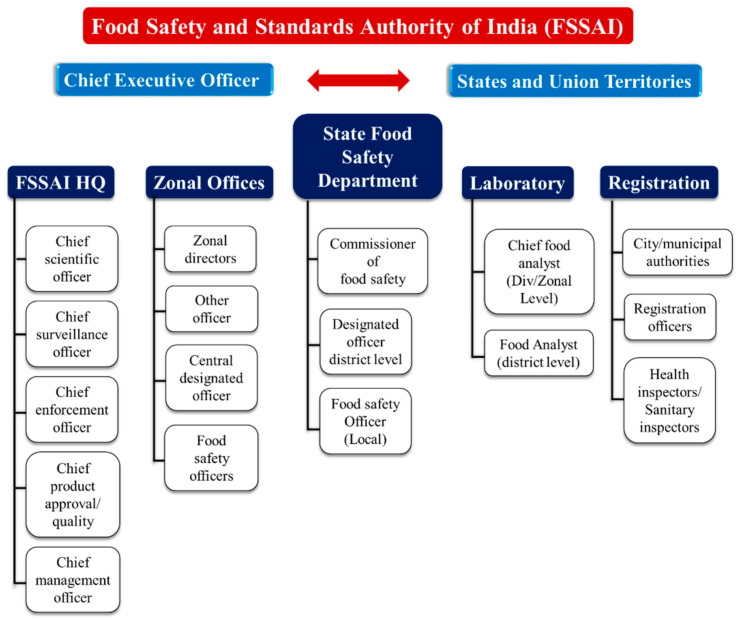
Food safety monitoring organizational structure of India.

In India, Food Safety and Standards Authority of India (FSSAI) is an autonomous statutory body established under the Food Safety and Standards Act, 2006 (FSS Act). The food control management is characterized as a process that involves planning, arranging, tracking, coordinating, and communicating in an organized way. It focuses on a wide range of risk-based government decisions and actions for public protection and quality of domestic produce, imported and exported food (domestic/international) [77]. The Food Safety and Standards Act of 2006 required anyone involved in the handling, manufacturing, delivery, or supply of food, no matter how small, to register. Figure 13 depicts a comprehensive outline of food control management in India.

### 4.3. Digitalization and Technological Advances in Food and Beverage Industries in China and India

The food and beverage industry’s volatility in COVID-19 has been exacerbated by rising agri-food sector demand. Consumers are far more discerning, willing to pay more for food that is safe, sustainable, and ethically produced [78]. Transparency in the agri-food sector, from production to consumption, is needed to meet these demands [79]. In the food sector, transparency and traceability are seen as the most significant differentiators between food brands [80,81]. The food and beverage business is being rapidly digitalized and this development is unlikely to slow down. This is no wonder that enterprises are adopting new technologies to boost their processes and competitiveness in an environment driven by smart systems, Internet of Things (IoT) devices, and automation. During the pandemic, the food distribution side of the market and how technology is taking it forward by new technologies such as smart sensors and interactive inventories, received a lot of attention in order to provide a consistent picture of a product’s lifecycle from design to production with operation [82]. Several studies [83,84,85] show that blockchain technology has the ability to revolutionize the agri-food supply chain structure. According to [86,87], blockchain technologies could be used to track food data and farm operations. This will facilitate the development of trust among the various stakeholders in the agri-food system [88].

According to Jiang [89], the usage of digital technologies in China has expanded since the pandemic, in line with the global pattern of digitalization. Novel prospects have emerged as a consequence of emerging technologies, new demands, and new customer preferences. Digital technology in pandemic detection and monitoring, as well as online learning, food and beverage delivery, co-working, and e-commerce, were found to be highly efficient in unpredictable COVID-19 like situations.

India’s food and beverage sector is going under extraordinary modifications, mainly through digitalization. One of the leading organizations in India, the “Chitale Group”, has implemented a traceability system through which monitoring of milk is accomplished right from purchase to the sale. A high quality standard is maintained across their supply chain, where breakdown at any point can be traced with the aid of digitalization and IoT, along with tracking the operation performances [90].

Nilesh Lele, the president of the “Association of Food Scientists and Technologists (India) Mumbai Chapter” in one of his interviews reported that the “dairy” sub-sector has been on the forefront of automation and digitalization. The feed and other items utilized in dairy are being automated and several dairies are going into automation in India, where the milk is now untouched by humans [90].

#### 4.3.1. Robotics

The world’s first “robot restaurant complex” started out in China’s Guangdong province by Qianxi Robot Catering Group. About 40 robots serve and cook over 200 meals at the kitchen. Customers place their orders with robot waiters, and their food is delivered by sky rail or on a tray directly to their table. The restaurant is symbolic of China’s rising robotics industry, which is worth USD 5.4 billion [91]. Due to the need to minimize physical interaction during the COVID-19 pandemic, robotics has grown to a new stage [92]. Since January, they have been used to disinfect clinics, serve meals to the sick, and patrol the streets. Any of these “Robocops” are capable of taking people’s temperatures and issuing public safety warnings, as well as reprimanding anyone found without a facemask in public. During the peak period of pandemic, those robots—and aerial drones—broadcasted “Please carry a mask, pay attention to personal safety, and avoid busy places” over loudspeakers in crowded cities.

Restaurants in India are attempting to reclaim normalcy by implementing automated and creative facilities [93]. Restaurants are inviting people for dine-in services now that the lockdown has been lifted in most Indian cities. Restaurants are implementing new and creative measures to make it “Safe-To-Eat-Out” for their customers once again, thus putting a priority on social distancing and hygiene. To minimize human touch, restaurants such as Robot (Porur) and Moti Mahal, Robot Themed Restaurant in Chennai, Robot Restaurant in Bangalore, and Robot Restaurant in Jaipur serve food with the help of robots. With food and mocktails, people can have an unforgettable robot experience. Cloves restaurant in Ahmedabad, Gujarat, created the first techno-dining experience in India. Food is delivered in covered bowls from the kitchen to the customer’s table via a food conveyor with no human interaction in between. Customers may pick up their own bowl with their table numbers written on it, which removes the need for stewards to handle food in between. Madras Canteen and Grill in Chennai uses a graphene-based nano disinfection solution to provide antiviral and antibacterial protection to its customers and employees 24 h a day, 7 days a week. Every day, all surfaces of the restaurant are disinfected, including the floors, walls, kitchen cutlery, counters, food service areas, toilets, lounge bar, smoking zones, and security area. Businesses have begun to rebound because of these interventions and developments. In the festive season, Dine out, a reservation and restaurant technology network, predicted a 70% recovery rate in Delhi, Bangalore, and Ahmedabad. Kolkata, Chennai, Jaipur, and Hyderabad followed with a 55% share [94].

#### 4.3.2. Drones and AI in China’s and India’s Food and Beverage Industry: Ensuring Transparency for the Customer

Delivery drones are a well-known artificial intelligence (AI) technology in China’s food and beverage industry [95]. These are meant to make meal delivery simpler in a world where an estimated 355 million people use food delivery apps. Drones, on the other hand, necessitate the establishment of air routes across the cities. The technology is in continuous advancement, which is causing a serious problem of brand rivalry. Who will build the longest roads in the countryside, and who will build the roads above Beijing? In China, the fight for automated food distribution has only just begun.

The monitoring of the food supply chain through AI implementation directly serves the interests of customers in China [96]. Mengniu Dairy is the second-largest dairy company in China, attempting to modernize the centuries-old industry by introducing AI into China’s food and beverage industry. The dairy industry, in particular, is extremely fragile and uninterrupted monitoring of a constant temperature level is a prerequisite. Mengniu has collaborated with Alibaba by implementing AI to examine the supply chain and decision making in site selection for products and milk production, as well as how to provide fast delivery at home. In future, Alibaba is planning to incorporate food tracking blockchain technology into its supply chain. Last year, the company conducted a traceability test using AI to monitor the source of a shipment of mangoes. Traditional approaches took 6 days and 18 h; remarkably, AI only took 2.2 s, respectively.

In 2020, Ele.me and Meituan Waimai accounted for more than 90% of the industry [97]. From 24 January to 2 February 2020, the transaction volume of JD Daojia, an online grocery store owned by JD.com, China’s second-largest e-commerce firm, increased by 374% year on year. Missfresh, a Beijing-based online fresh food e-commerce website, saw trading volume rise by 321% from 24 January to 28 January compared to the previous year [98]. Daily new users on major fresh food e-commerce and online to offline (O2O) platforms have increased by 50% to 200%, and transaction volume has increased three to four times year on year on these platforms. According to the survey, the Chinese food delivery market was worth CNY 653.6 billion in 2019, up 39.3% from 2018. At the same time, the food delivery industry’s penetration rate rose by 3% to 14% in 2019, compared to 13% in 2018. There were approximately 460 million Chinese food delivery customers as of the end of 2019, accounting for 50.7% of the country’s 900 million Internet users and 53.9% (848.43 million) of the country’s urban permanent population [97].

In India, with a land area of 3.28 million square kilometers, sanitizing and disinfecting was a significant challenge and danger for public sanitation workers who used a manual spraying technique. To minimize the risk of manual spraying workers and their families contracting the virus, automated sanitation using drones were introduced. In comparison to manual spraying, which takes an hour, these drones can complete the task in 15 min [99]. Padmanathan et al., 2019, used an advanced inter-disciplinary approach to investigate the cultural, economic, environmental, technological, and social sustainability of human beings. The authors of that study concluded that any new business models for different industries must counter the defiance that exists in a variety of rural settings [100]. As a result, Garuda Aerospace, an ISO 9001 start-up company, launched “Corona Killer” to provide drone-based disinfection services. These automated disinfecting drones can spray disinfectants from a height of 3 to 450 feet, covering large distances in a short amount of time without the need for public workers to be present. These drones were used for the Swaach Bharat campaign, which aims to keep India clean on a regular basis and prevent the spread of COVID-19 and other viral and infectious diseases. Cities such as Chennai, Chandigarh, Varanasi, Raipur, and Bhopal are examples.

The Indian food industry has advanced by integrating cutting-edge technology into its day-to-day operations. Companies that use artificial intelligence have a major competitive advantage over those that do not. Companies such as Zomato, Swiggy, and Foodpanda, have embraced AI to provide the best possible experience. Other online food delivery companies are using artificial intelligence to improve customer experiences, fueled by their development. In the food industry, artificial intelligence has proven to be useful in a variety of ways [101].

India’s food processing industry was projected to hit USD 4 billion by 2020. Every new startup aspires to be the market leader by using artificial intelligence in some way. Food delivery sites like Zomato and Swiggy have seen rapid growth in their user bases and the effect of AI on the food industry has been extremely positive [101].

### 4.4. National Strategies, Policies, and Responses in COVID-19 Crisis

While public attention has been drawn to problems in global food supply chains, little is known about the impact of COVID-19 on agricultural production; most current research focuses on logistics and distribution [102,103]. In reality, China’s experience has revealed that certain COVID-19 countermeasures have caused disruptions in economic development process. This poses a significant risk to the long-term food supply. Such cases of production sabotage can be found not only in China, but also in India. The national strategies and responses for the food and agri-allied market and the following strategies which have been established so far in both nations are shown in Table 1 and Table 2, respectively.

## 5. Future of Chinese and Indian Food and Beverage Industry Post-Pandemic

Due to the COVID-19 crisis, China’s food and beverage industries bore the brunt of demand–supply challenges for many months. The pandemic has had a significant impact on the global supply chain, food service market, commodity prices, and demand for both essential and non-essential goods, particularly after some of China’s major cities were put on lockdown. Companies in China’s food and beverage sector have begun to restart operations as the country recovers from the coronavirus outbreak that lasted months. However, the country’s food and beverage market is still far from returning to normalcy; much has changed in the consumer’s attitude toward the F&B sector since the crisis. The industry appears to be dancing to a different tune, from the introduction of automatic, contactless payments despite increasing food safety issues to secure online shopping and offline food distribution using “no touch” technology. As a result, China’s food and beverage firms are introducing new programs to counter the massive change in customer buying habits. The following are some of the developments that F&B companies around the world should expect in the near future, based on changing customer behavior in China.

With over 7.3 million workers, the food and beverage industry accounts for 3% of India’s GDP and is the country’s single largest employer-generating sector. The nationwide lockdown sent the restaurant industry into a tailspin, with some estimates predicting that nearly a quarter of all restaurants nearly closed by the end of 2020. According to the National Restaurant Association of India, it was predicted that India’s USD 50 billion restaurant industry will lose USD 9 billion in 2020 (NRAI). However, the real estimates are yet to be available.

Since the lockdown was lifted, the industry has been adjusting and innovating to resolve these obstacles and regain profitability. In order to gain customer trust and increase sales, the sector is introducing new service offerings and COVID hygiene protocols.

### 5.1. Hygiene Standards

In China, consumers are becoming increasingly aware of the value of hygiene and food safety. To improve visibility and transparency in their supply chains, food and beverage manufacturers around the world should consider technological advances like blockchain.

On the other hand, India’s food safety and hygiene has seen exponential growth in recent years. In the food and beverage industry, temperature control, regular cleaning, the use of masks, and safe packaging systems have become the new norms. Furthermore, health and safety auditing firms are constantly being used to ensure compliance and boost customer trust. Currently, such audits are not required in India, though the FSSAI has released a 48-point checklist on hygiene ratings for restaurants to adopt, with the goal of developing more rigorous hygiene standards and safety auditing protocols in the future [97].

### 5.2. Contactless Solutions

In China, with most food service firms halting operations due to the coronavirus outbreak, canned foods have seen a surge in demand in China in recent months.

Both in China and India, a focus on health, safety, protection, contactless solutions, and self-service stations will be the new normal. Delivery robots, digital menus, and in-app ordering are some of the most common contactless solutions. Face recognition and contactless biometrics are being used in the hospitality industry to enable guests to self-check-in and check-out, unlock rooms, and activate elevators using facial scans [104,105].

To regain market momentum, the industry has used the downtime caused by the pandemic to invest in customer-centric technologies. For the industry to promote automated conversational interaction between guests and service workers, contactless guest engagement has become a priority. Touchless solutions based on AI and digital payment options are likely to become the new norm in the hospitality industry in the post-Covid era, changing industry standards [104].

### 5.3. At-Home Experiences

Online shopping is becoming more common in China as a result of the pandemic, with a greater number of elderly people using online shopping platforms than ever before [106]. An easy-to-use interface, simple images, and easy-to-read product descriptions are needed to fuel the trend and increase the popularity of food delivery among this demographic [107].

On the contrary, to attract consumers, a growing number of food and beverage companies have begun to deliver pre-packaged or customized “At Home” experiences in India [108]. Previously, a limited number of players as a niche product were pursuing this trend, but since the pandemic, these interactions have become a common service category within the industry. It is expected that a substantial portion of the population will use this service long after the pandemic has ended to cope with the fear of another outbreak. Private catering services with the option of “cooking at home” ingredient packages are available from most hospitality brands [104].

### 5.4. Vegan and Healthy Food Brands

In China and India, there has been a significant change in consumer preferences for vegan and organic foods [23,109]. Many businesses opened in late 2019 and early 2020 with an emphasis on organic, farm-to-table vegan menus. However, with the global success of COVID-19, this pattern is likely to become a way of life for many in the coming years. Given the rising number of coronavirus cases in the world, the numerous health- and immunity-boosting benefits of an organic, vegan diet are becoming increasingly common. As a result, “vegan only” restaurants and labels marketing plant-based products are projected to grow in popularity. Given that the Indian diet is predominantly vegan-friendly, most consumers will find the change and adoption to be fast and painless [104].

## 6. Conclusions

This research examined the impact of the pandemic, as well as the strategies and policies that dealt with those impacts in the food and beverage industry in China and India. Our investigation revealed that COVID-19 during 2019–2020 and currently ongoing has affected various factors in the food and beverage industry in China and India, for instance loss of enterprises, growth in revenue (%), total profit (%), gross value added, and growth value of exports (%). In addition, the negative and positive impact consensus was drawn from the investigation, where negative impacts represented several factors as mentioned in RO4 and positive impacts in RO5. These impacts will no doubt continue for a medium to long term period in China and India. These consequences are likely to include supply chain restructuring based on online trading, as well as a fall in the industry’s contribution to GDP in both nations. This study holds a high potential in delivering a better understanding of COVID-19 impacts on China and India’s food and beverage industry. As per the examined results, China and India witnessed supply chain disruptions causing decline in revenue generation in the F&B industry. In addition, due to movement restrictions, the manufacturing as well as domestic and international trade was severely affected in both nations, causing serious falls in the year-on-year growth in gross value added (GVA) and growth value of export. However, the food and beverage industries in China and India are undergoing a digital transformation. Services will become more personalized and customer-focused, with more creative service offerings. Due to stringent policies, prompt responses and technological advancements during COVID-19, health and safety will be highly strengthened and operations will become less labor-intensive in both nations. In the post-Covid era, such developments will boost the customer experience and set new industry standards.

In short, the present study holds a high potential for the scientific readers, policy makers, government administrative and investigation agencies, agricultural ministries and committees to better understand the ongoing COVID-19 impacts on the F&B industry and the responsive measures undertaken by China and India. This study will be highly beneficial in employing strategies, and responsive measures followed by two Asian giants in future unanticipated events such as COVID-19.

## Figures and Tables

**Table 1:** China’s strategies and response towards for sustainable food processing industry during COVID-19.

Date	Entities	Strategies	Response
19 January	MARA	Urgent notice on ensuring vegetable production and supply during COVID-19 epidemic	■ Strengthen production guidance ■ Help match supply with demand ■ Smooth transport channels
1 February	MC	Notice on further ensuring supply of life necessities (inc. grain and edible oil, meat, vegetables etc.)	■ Take quick actions to unblock transport channels from distribution centers to retail terminals ■ Check the number of necessity inventories and distribution methods ■ Stabilize the social expectation and increase public confidence
4 February	MARA	Urgent notice on maintaining livestock and poultry industry in operation and ensuring supply of meat, eggs, and milk	■ Do not block transport vehicles for livestock and poultry products ■ Do not close slaughter house ■ Support enterprises to resume work
6 February	MC	Notice on ensuring delivery and distribution of life necessities during COVID-19 epidemic	■ Help enterprises to increase efficiency in transportation of life necessities ■ Help enterprises to solve difficulties and problems in circulation
11 February	MC	Notice on further supply of life necessities in major cities	■ Increase inventory of life necessities ■ Establish and improve the emergency distribution system ■ Maintain market orders
11 February	MARA	Notice of ensuring agriculture products sales in poor stricken regions during COVID-19 epidemic	■ Coordinate with e-commerce enterprises to open special online sales channel ■ Give credit priority, subsidy loan interests ■ Quick sampling inspection on the quality and safety of agriculture products
14 February	MC	Notice on further strengthening linkages between agriculture demand and supply during COVID-19 epidemic	■ Increase commercial inventory by providing subsidies and interest discounts ■ Reducing fees, setting up special sales zones, and providing convenience certificates for sellers
14 February	MC, MF	Urgent notice on enhancing collaboration and coordination between agriculture and commercial sectors to improve the food supply chain during COVID-19 epidemic	■ Arrange a certain percentage of funds to maintain supply ■ Allow flexible use of funds ■ Simplify the procedures of fund appropriation
14 February	MF, MARA	Notice on ensuring stable production and adequate supply of agricultural products during COVID-19 epidemic	■ Reduce or waive the guarantee fees of agriculture credits ■ Distribute agricultural production disaster relief funds as soon as possible ■ Increase subsidies for cold storage and preservation facilities
16 February	MARA, NDRC, MT	Urgent notice on alleviating current practical difficulties and speeding up the resumption of breeding industry	■ Release more public corn storage ■ Open green channels for feed production ■ Enhancing collaboration between enterprises and the banks
21 February	MC	Notice on overall planning and management of life necessities supply	■ Coordinate well with key enterprises undertaking emergency supply tasks ■ Provide innovative supply modes such as (central purchasing and distribution by community online order and contactless delivery) ■ Simplify the approval process of resuming work
25 February	MC	Notice on popularizing the best practices of ensuring life necessities supply during the COVID-19 epidemic	■ Centralized purchasing and distribution for closed communities ■ Contactless delivery ■ Developed electronic maps to provide accurate cells information for the residents ■ Provide various standardized food combos
2 March	LGCC COVID-19	Guidelines for agricultural production in spring planting season	■ Maintain the planting areas of grain crops ■ Differentiated resuming spring planting majors in accordance with local risk levels ■ Ensure smooth transportation and sufficient supply of agricultural material ■ Provide online services of agricultural guidance
13 March	MARA	Notice on further simplifying certification and approval process to speeding up the resumption of agricultural enterprises	■ Simplify examination and approval procedures ■ Temporarily exempt from onsite inspection processes ■ Compress the approval time by over one third ■ Expand the scope of online approval
19 March	MARA, MF, CBIRC	Notice on further strengthening supports to ensuring stable production and supply of pork	■ Lower the threshold for loan interest subsidy from 5000 pigs to 500 pigs ■ Extend the valid date of policy-based agricultural credit loan to 31 December 2020 ■ Relax the standard of non-performing loan ratio for hog breeding

Abbreviations: Ministry of Agriculture and Rural Affairs (MARA), Ministry of Commerce (MC), Ministry of Finance (MF), National Development and Reform Commission (NDRC), Ministry of Transport (MT), China Banking and Insurance Regulatory Commission (CBIRC), Leading Group of the Central Committee on the Response to COVID-19 (LGCC COVID-19).

**Table 2:** India’s strategies and response towards for sustainable food processing industry during COVID-19.

Date	Entities	Strategies	Response
24 March	MAFW	Launched new features of the electronic National Agriculture Market (e-NAM) platform	■ In order to reduce the need to physically travel to APMC markets for selling crops
25 March	MOHA	Issued a notice information to states and UTs	■ Transportation of animal feed and fodder was considered an essential service and would thus be exempted from any inter-state restriction under the 2005 Disaster Management Act ■ Inter-state movement of harvesting and sowing machinery ■ Special railway parcel trains for the transportation of essential items, including food products, in small parcel sizes
25 March	NDDB	Urged all milk co-operatives	■ Ensure supply of milk and milk products, against the background of co-operatives such as the Karnataka Cooperative Milk Federation stopping sales of milk to neighboring state
26 March	APMC	Took proactive measures	■ Ensure supply of essential vegetables and fruits is not hit during the lockdown period
26 March	C&SG	Made efforts to maintain the operation of distribution channels	■ Almost 1900 vegetable markets retook their operations in order to ensure a smooth supply of fruit and vegetables
31 March	MOS	Issued specific guidelines to main ports	■ Exemptions and reductions of penalties, demurrages charges, and other port fees for traders in relation to any potential delay in cargo port operations
27 March	CG	Extend the current Foreign Trade Policy	■ All the existing schemes under the current policy will be extended over 2020–2025 period
16 April	GOI	Govt allowed e-commerce, agri industry to resume from April 20	■ To reduce the distress caused to millions of people because of the lockdown
15 May	FM	Unveiled third set of economic stimulus	■ Focusing on agriculture and allied activities—dairy, fisheries, food processing, animal husbandry

Abbreviation: Ministry of Agriculture and Farmers Welfare (MAFW), Ministry of Home Affairs (MOHA), Union Territories (UTs), National Dairy Development Board (NDDB), Agriculture Produce Marketing Committee (APMC), Central and State Governments (C&SG), Ministry of Shipping (MOS), Central Government (CG), Government of India (GOI), Finance Minister (FM).

## Data Availability

The data presented in this study are available on request to authors.
